# Endogenous glycosaminoglycan anticoagulation in extracorporeal membrane oxygenation

**DOI:** 10.1186/s13054-014-0636-4

**Published:** 2014-11-21

**Authors:** Graeme MacLaren, Paul Monagle

**Affiliations:** Cardiothoracic Intensive Care Unit, National University Health System, 5 Lower Kent Ridge Road, 119074 Singapore, Singapore; Paediatric Intensive Care Unit, The Royal Children’s Hospital, Flemington Road, Parkville, VIC 3052 Australia; Department of Paediatrics, The University of Melbourne, Flemington Road, Parkville, VIC 3052 Australia; Clinical Sciences Research, Murdoch Childrens Research Institute, Flemington Road, Parkville, VIC 3052 Australia; Department of Haematology, The Royal Children’s Hospital, Flemington 5 Road, Parkville, VIC 3052 Australia

## Abstract

A heparin-like effect was recently described in infants, children, and adults receiving bivalirudin while supported on extracorporeal membrane oxygenation following cardiopulmonary bypass. This is most likely due to endogenous release of glycosaminoglycans from vascular endothelium and mast cells and is associated with longer duration of extracorporeal membrane oxygenation and an increased incidence of sepsis. Further investigation into this effect should include patients without recent cardiopulmonary bypass, exclude the presence of covalent antithrombin-heparin complexes, and employ a variety of different heparinases for thromboelastography. The phenomenon may partially explain the heterogeneity of anticoagulation requirements in patients on extracorporeal life support.

## Introduction

Extracorporeal membrane oxygenation (ECMO) has been used for over 40 years in patients with refractory respiratory failure or shock. Over this time, there have been continuous modifications and improvements in both the circuitry and clinical management of ECMO. However, one perennial problem has been the need to anticoagulate blood passing through the circuit. Finding a balance between the risks of bleeding and clot formation is arguably the most challenging problem in ECMO today, especially in complex, vulnerable populations such as infants, multitrauma patients, or when ECMO is deployed after cardiopulmonary bypass (CPB). Unfractionated heparin (UFH) remains the most commonly used anticoagulant for ECMO. Heparin is a naturally occurring glycosaminoglycan (GAG) that is usually bound to endothelium but can be secreted into the bloodstream. Other GAGs, including heparan sulphate, chondroitin sulphate, and dermatan sulphate, are known to prolong anticoagulation tests and thus exhibit a heparin-like effect (HLE), although they are not usually used as therapeutic agents.

## Commentary

In this issue of *Critical Care*, Ranucci and colleagues [1] demonstrate a novel phenomenon in a cohort of post-cardiotomy patients receiving bivalirudin on ECMO. Based on thromboelastography with and without heparinase, the authors documented HLE in over half the cohort, even although none of the patients received UFH after the conclusion of CPB. HLE was associated with a longer duration of ECMO and an increased incidence of sepsis. The authors speculated that the HLE was due to release of native heparinoids from the glycocalyx and mast cells.

There are many aspects to consider with these intriguing new findings. A variety of heparinases are commercially available, but the specifics of the ones used in this study were not provided, so it is unknown whether the heparinase used was specific for cleaving heparin and heparin sulphate or whether it cleaved a wider range of GAGs such as dermatan or chondroitin sulphates. The nature of the molecule causing the HLE thus remains to be elucidated. The authors may be correct in their hypothesis that GAGs released from the vascular endothelium were responsible, but an increase in GAGs may also have been secondary to increased production in other tissues or because of inadequate hepatic clearance. It is equally unclear whether they were released as a consequence of sepsis or as a result of damaged endothelium which might later render the patients more vulnerable to sepsis. In other words, were GAGs the cause of the apparent differences in clinical outcome, a consequence, or completely unrelated?

If the HLE was unrelated to endogenous GAG release, what other reasons might explain the authors’ findings? All of the patients in the study received UFH during CPB. Given that the HLE was seen predominantly in the first 7 days post-bypass, one possible explanation is that there was a reservoir of heparin in an extravascular compartment which later redistributed into the blood after CPB was completed. In patients who have CPB with UFH, there is often a rebound of measures of UFH activity in the 24 hours following reversal with protamine. However, after following a control group who were not on ECMO, the authors found nothing to support this hypothesis, so attributing HLE to heparin rebound appears unlikely.

A more intriguing alternative is that the persistent effect over the first week after CPB represented a residual covalent antithrombin-heparin complex (ATH) formed during the high-concentration UFH of CPB. ATH may not be well reversed by protamine and is known to have a very long half-life [2,3]. ATH most likely forms under the conditions of CPB, but little research has been done to establish how frequently or to what extent it occurs in patients. A simple means of investigating this further would be to repeat the study in patients who are on ECMO for reasons unrelated to CPB, where no heparin was used prior to ECMO. Alternatively, ATH levels could be measured and correlated to the R-time on thromboelastography.

This study has a number of limitations, which are acknowledged by the authors. It was a small, retrospective, single-center study with an extremely heterogeneous population combining neonatal, pediatric, and adult patients. A variety of ECMO machines were used, the surface coatings of which can elicit different pathophysiological responses from vascular endothelium. Additionally, the study described neither where the blood samples for the thromboelastography were taken from nor whether the samples were taken from heparinized lines, raising the possibility of heparin contamination. Despite these limitations, it appears probable, given the data presented, that the authors have demonstrated a potentially important new finding with clinical implications that mandate further research.

## Conclusions

The uncertainty surrounding optimal anticoagulation practices for ECMO was recently highlighted by the Extracorporeal Life Support Organization in its anticoagulation guidelines [4]. Despite decades of research, the complex interactions of coagulation and inflammation pathways during ECMO are poorly understood. The findings, underlying hypotheses, and potential clinical implications of the present study will be of interest to ECMO clinicians. Further research from other centers and in non-CPB patients is warranted to confirm whether the phenomenon is reproducible and, if so, to determine the mechanism. A hitherto undetected HLE might go a considerable way toward explaining the heterogeneity of anticoagulation requirements in ECMO patients. By elucidating the mechanism of HLE, we will not only learn more about the pathophysiological role of GAGs in critical illness, we may yet make anticoagulation in ECMO safer and simpler.

## Abbreviations

ATH: Antithrombin-heparin complex
CPB: Cardiopulmonary bypass
ECMO: Extracorporeal membrane oxygenation
GAG: Glycosaminoglycan
HLE: Heparin-like effect
UFH: Unfractionated heparin
